# Factors associated with seasonal influenza vaccine uptake among children in Japan

**DOI:** 10.1186/s12879-015-0821-3

**Published:** 2015-02-18

**Authors:** Aiko Shono, Masahide Kondo

**Affiliations:** Department of Public Health and Epidemiology, Faculty of Pharmaceutical Sciences, Meiji Pharmaceutical University, 2-522-1 Noshio, Kiyose, Tokyo 204-8588 Japan; Department of Health Care Policy and Health Economics, Faculty of Medicine, University of Tsukuba, 1-1-1 Tennoudai, Tsukuba, Ibaraki 305-8577 Japan

**Keywords:** Seasonal influenza vaccine, Self-paid vaccine, Voluntary vaccination, Children

## Abstract

**Background:**

Seasonal influenza vaccine was once part of the routine immunization schedule that is routinely offered to all children in Japan, but it is now excluded from the schedule. This study aimed to investigate factors influential to parents’ decision to have their children receive seasonal influenza vaccine, as well as types of seasonal influenza vaccine information that is given to parents.

**Methods:**

We conducted a cross-sectional online survey of 555 participants who have at least one child younger than 13 years of age. Respondents were asked to categorize the history of influenza vaccination of their youngest child as either ‘annual’ , ‘sometimes’ , or ‘never’. Participants were also asked about potentially influential factors in their decision to have their children receive a seasonal influenza vaccine.

**Results:**

A total of 75% of respondents answered that their youngest child had received a seasonal influenza vaccine, and 57% of respondents answered that their child receives the vaccine every year. The higher income group was more likely than the lowest income group to have a history of influenza vaccine uptake. A recommendation from a pediatrician or school/nursery to have their child vaccinated was also positively associated with a history of influenza vaccine uptake. The most common reason for a pediatrician’s recommendation was ‘it leads to milder symptoms if infected’.

**Conclusions:**

The main finding of the study is a significant association between household income and influenza vaccination of the youngest child in the household. We also found that cost could be a barrier to vaccinating children in low income households and that information from pediatricians and schools/nurseries could motivate parents to have their children vaccinated.

## Background

In Japan, routine vaccination is defined by the Preventive Vaccinations Act, while voluntary vaccination is not regulated by the law. Although voluntary vaccines are approved by the Ministry of Health, Labour and Welfare under the Pharmaceutical Affairs Law, they are not vaccines that are required by Preventive Vaccinations Act [1,2]. In recent years, some vaccines such as *Haemophilus influenzae* type b (Hib) vaccine and pediatric pneumococcal conjugate vaccine have been added in the routine immunization schedule [3]. Seasonal influenza vaccination is designated as a routine vaccination for the elderly but voluntary vaccination for children [2]. Historically, seasonal influenza vaccination was part of the routine immunization schedule offered to schoolchildren, and the vaccine was administered by mass vaccination of schoolchildren in classrooms. However, in 1994 it was excluded from the routine immunization schedule as a result of a negative campaign against influenza vaccination that began in the late 1980s that questions vaccine efficacy and emphasizes vaccine risks and adverse events. As a result of this change, the decision to vaccinate children against seasonal influenza has been left to the discretion of parents, who must pay for the vaccine out-of-pocket [1,4]. In the 2010/11 influenza season the coverage rate of seasonal influenza vaccine among children was estimated to be 11% in children younger than 1 year old, 70% in those 1–6 years old, and 58% in those 6–13 years old [5].

Many studies have shown the usefulness of influenza vaccination for the prevention of influenza infection and disease. Some studies report that immunizing children can protect not only the children but also the community from seasonal influenza [6]. Mass vaccination of schoolchildren in Japan is being revalued for its protective impact on influenza-associated mortality among young children and older persons, as well as reducing class cancellation in schools [7-9]. Many advisory groups, including the Japan Pediatric Society, recommend that healthy children aged 6 months and older receive the influenza vaccine [10-12]. This study aims to investigate factors influential to parents’ decision to have their children receive the seasonal influenza vaccine in Japan.

## Methods

Respondents were recruited from a registered online survey panel of a web-based private survey company in January 2013. Parents who had at least one child under 13 years of age were asked to participate in the full survey (approximately n = 6,000). Recruitment ceased when the number of respondents reached the target of 555. This number was estimated from the main hypothesis that the influenza coverage rate is 65% for the higher income group, and 55% for the lower income group from data indicating that influenza vaccination coverage among children is around 60%. The significance level α was set at 0.05, and the power at 0.55. Therefore it was necessary to study about 250 respondents from each income group [13]. We then employed a survey company that ultimately collected 555 responses. Because this survey is of a non-random sample, the geographic structure of the child population distribution of the participants was adjusted to be similar to nationwide statistics in Japan. Household income of respondents was also adjusted to fit the distribution of household incomes of families with children based on nationwide statistics [14,15].

For factors that are potentially influential to childhood vaccine uptake, we inquired about respondents’ education, marital status, number of children, household income, mother’s employment, youngest child’s history of influenza and history of influenza vaccine receipt, and recommendation(s) from a pediatrician or school/nursery. The three categories of history of influenza vaccine were ‘annual’ , ‘sometimes’ , and ‘never’. The four categories of recommendation on influenza vaccination from a pediatrician were shown as ‘yes’ , ‘either vaccination or not is acceptable’ , ‘recommended no vaccination’ , or ‘did not make a recommendation’ , and respondents were asked to choose one of the four. ‘Either vaccination or not is acceptable’ means that either option was equally acceptable, and the pediatrician left the decision to the parents. ‘Recommended no vaccination’ means that the pediatrician said the child should not receive the vaccine. We also inquired about the types of information offered by pediatricians when recommending influenza vaccination. For the recommendation from a school/nursery, the four categories were ‘yes’ , ‘either vaccination or not is acceptable’ , ‘child does not go to any school/nursery’ , and ‘did not make a recommendation’.

Ordered logit models were applied to investigate associations between the history of influenza vaccination and possible factors influencing vaccination. The dependent variable, the history of influenza vaccination of the youngest child, was coded as 2 if parents reported ‘annual’ , as 1 if ‘sometimes’ , and as 0 if ‘never’. Each variable was first examined by bivariate analysis. Variables that could possibly influence vaccination included: the number of children; household income (unit: 10,000 yen [US$112]); mother’s employment (full time job, part time job, self-employed, or unemployed); years of education of the respondent; marital status of the respondent (married or other); prior diagnoses of influenza in the youngest child (yes [including maybe] or no/do not remember); and recommendation from the pediatrician (yes, either vaccination or not is acceptable, recommended no vaccination, or did not make a recommendation) and the school/nursery (yes, either vaccination or not is acceptable, did not make a recommendation or the child does not attend any school). Although we considered the age of the child as a possible confounder, we did not find any strong associations with other variables. Multivariate analysis was then conducted to adjust for the effects of other variables. We considered differences significant at *p* < 0.05. The survey protocol was approved by the Ethical Committee of Meiji Pharmaceutical University. Informed consent was obtained from all respondents.

## Results

Respondents’ characteristics are shown in Table 1. More than half of the respondents were female (59%), 57% were aged 30–39 years, and the vast majority (97%) were married. The mean number of years of education of the respondent was 15 years, and the most common educational background was a bachelor’s degree (42%). The average annual household income was 6.7 million yen (US$75,506).

**Table 1:** **Respondent characteristics**

**Variables**	**N (%)**
Gender	
Female	325 (59)
Age (years)	
≤19	0 (0)
20 − 29	40 (7)
30 − 39	314 (57)
40 − 49	176 (32)
≥50	25 (5)
Marital status	
Married	540 (97)
Other	15 (3)
Number of children	
1	246 (44)
2	265 (48)
3	38 (7)
≥4	6 (1)
Schooling of the respondent (years) (mean, SD)	14.7 (1.9)
Annual household income (million yen^a^) (mean, SD)	6.72 (3.62)
Annual household income by quintile group (million yen) (mean, SD)	
Lowest	2.71 (0.86)
Second lowest	5.02 (0.50)
Middle	6.50 (0.00)
Second highest	8.37 (0.80)
Highest	13.29 (1.83)
	N = 555

^a^US$1 = 89 yen (as of January 2013).

A total of 75% of respondents answered that their youngest child had received a seasonal influenza vaccine, and 57% of respondents answered that their youngest child receives the vaccine every year (Table 2). During the 2012/13 season, 58% of children aged < 6 years old, and 64% of those aged 6–13 years old in this study received a seasonal influenza vaccine. Less than half of the respondents (43%) reported that their youngest child had a prior diagnosis of influenza. A majority of respondents (60%) answered that the child’s mother was unemployed. A total of 14% of respondents received a recommendation from a pediatrician, and 13% of respondents received a recommendation from a school/nursery (Table 2).

**Table 2:** **Factors that may influence seasonal influenza vaccination of children**

**Variables**	**N (%)**
History of influenza vaccination^a^	
Every year	314 (56.6)
Sometimes	104 (18.7)
Never	137 (24.7)
Vaccination coverage	
<6 years old	171 (58.2)
6–13 years old	167 (64.0)
Child had a prior diagnosis of influenza	
Yes	239 (43.1)
No/do not remember	316 (56.9)
Mother’s employment^b^	
Yes	220 (39.7)
Unemployed	334 (60.3)
Pediatrician’s recommendation	
Yes	80 (14.4)
Either vaccination or not is acceptable	13 (2.3)
Recommended no vaccination	12 (2.2)
Did not make a recommendation	450 (81.1)
Recommendation from school/nursery^b^	
Yes	71 (12.8)
Either vaccination or not is acceptable	19 (3.4)
Did not make a recommendation	315 (56.8)
Child does not attend school	149 (26.8)

^a^Vaccination means influenza vaccination on this table.

^b^The total does not sum to 555 because of missing values.

In bivariate analysis, years of schooling of the respondent, and previous influenza diagnosis in the child were positively associated with a history of influenza vaccination for the youngest child (Table 3). A recommendation from a pediatrician or a school/nursery was positively associated with a history of influenza vaccination for the youngest child, compared with ‘did not make a recommendation’. The higher income group was more positively associated with a history of influenza vaccine uptake than the lowest income group. Conversely, compared with ‘did not make a recommendation’ , ‘recommended no vaccination’ by a pediatrician and having a child not go to a school/nursery showed significant negative correlations with a history of influenza vaccination for the youngest child.

**Table 3:** **Ordered logit model analysis of factors affecting the history of influenza vaccination of the youngest child**

**Characteristic**		**Bivariate model**				**Multivariate model**			
		**Coefficient**	***p*** **value**	**95% CI** ^**a**^		**Coefficient**	***p*** **value**	**95% CI**	
Number of children		0.07	0.58	−0.18	0.32	0.12	0.38	−0.15	0.38
Annual household income (quintile)	Lowest	Reference							
	Second lowest	0.39	0.09	−0.06	0.84	0.26	0.31	−0.24	0.76
	Middle	0.73	0.01	0.16	1.30	0.59	0.07	−0.04	1.22
	Second highest	0.79	0.00	0.31	1.27	0.64	0.03	0.07	1.20
	Highest	0.90	0.00	0.36	1.45	0.64	0.05	0.01	1.27
Mother’s employment	Unemployed	Reference				Reference			
	Yes	0.19	0.27	−0.14	0.52	−0.24	0.21	−0.62	0.14
Schooling years of respondent (years)		0.11	0.01	0.03	0.20	0.08	0.10	−0.02	0.19
Marital status of respondent	Other	Reference				Reference			
	Married	0.56	0.27	−0.43	1.54	−0.55	0.37	−1.73	0.64
Child had a prior diagnosis of influenza	No/do not remember	Reference				Reference			
	Yes	0.46	0.01	0.13	0.79	0.27	0.17	−0.11	0.65
Pediatrician’s recommendation	Did not make a recommendation	Reference				Reference			
	Yes	1.50	0.00	0.90	2.10	1.43	0.00	0.78	2.07
	Either vaccination or not is acceptable	1.14	0.08	−0.14	2.43	1.33	0.06	−0.03	2.69
	Recommended no vaccination	−2.71	0.00	−4.22	−1.19	−2.84	0.00	−4.44	−1.24
Recommendation from school	Did not make a recommendation	Reference				Reference			
	Yes	1.18	0.00	0.55	1.81	0.99	0.00	0.32	1.67
	Either vaccination or not is acceptable	0.63	0.20	−0.33	1.58	0.11	0.84	−0.91	1.13
	Child does not attend school	−0.97	0.00	−1.36	−0.58	−0.86	0.00	−1.30	−0.41
	Number of observation = 553								
	Pseudo R2 = 0.104								

^a^CI: Confidence interval.

In the multivariate model, the higher income group was more likely than the lowest income group to have a history of influenza vaccine uptake (Table 3). Like the bivariate analysis, a recommendation from a pediatrician or school/nursery was significantly associated with a history of influenza vaccination of the youngest child, compared with ‘did not make a recommendation’. Additionally, ‘recommended no vaccination’ by a pediatrician and having a child not go to a school/nursery showed significant negative correlations with the history of influenza vaccination for the youngest child, compared with ‘did not make a recommendation’. However, compared with ‘did not make a recommendation’, a pediatrician’s advice of ‘either vaccination or not is acceptable’ was not significantly associated with a history of influenza vaccination of the youngest child.

Of the reasons for a pediatrician to recommend influenza vaccination, the most common was ‘symptoms are mild if infected’ (47%) followed by ‘to prevent influenza infections’ (24%) (Table 4). Reasons for pediatricians to not recommend an influenza vaccination were because of an individual child’s characteristics such as an egg allergy; however, recommendations against vaccination were few.

**Table 4:** **Pediatrician reason for recommending influenza vaccination**

	**N (%)**
Symptoms are mild if infected	35 (47)
To prevent influenza infections	18 (24)
Child has an underlying disease	10 (13)
For communal living	8 (11)
Never heard the reason	2 (3)
Others	2 (3)
	N = 75

## Discussion

The aim of this study was to explore factors influencing parents’ decisions to have their children receive the seasonal influenza vaccine in Japan. The main finding of the study is a significant association between household income and influenza vaccination of the youngest child in the household. Socioeconomic determinants have been explored as factors to explain influenza vaccine uptake in many countries [16]. In the Japanese setting, because seasonal influenza vaccination for children is voluntary, parents must pay for the vaccine out-of-pocket. The recommendation in Japan is for children to receive influenza vaccine twice during each winter season [11]. According to a survey by a private company in 2008, the average price per child is 2,702 yen (US$30) for the first shot and 2,379 yen (US$27) for the second [17]. Therefore, the associated cost for influenza vaccination could be a heavy burden on low income households. Prior research showed that preventive medicine, including influenza vaccination, is favored by higher socioeconomic groups [18,19]. Although we cannot definitively state that our results showed inequity, we can say that cost could be a barrier to vaccinating children in low income households. Therefore, financial support for influenza vaccination for low income households might improve children’s influenza vaccination coverage [20]. Moreover, if unvaccinated children were infected with influenza, they may have more severe disease and be more likely to visit a physician than vaccinated children [21]. The burden of disease could be heavy on households as well as children.

Other findings of this study were that parents given a recommendation from a pediatrician and/or school/nursery were more likely to have their youngest child vaccinated. Information from pediatricians and schools/nurseries as trusted information sources could motivate parents to have their children vaccinated [22]. However, in this study parents reported that pediatricians did not always emphasize vaccine efficacy to protect against influenza. These results might reflect what Hirota & Kaji [4] stated: ‘many physicians and pediatricians usually make apologies when administering influenza vaccine, explaining that “Every vaccine recipient cannot necessarily avoid contracting influenza”.

We recognize some limitations to this study. One limitation is the fact that the number of respondents is a relatively small number (555). Additionally, this was a web-based survey; thus, respondents were limited to the population who can access the Internet. This could cause selection bias. However, in Japan over 90% of the population aged 20–50 years could access the Internet in 2011 [23]. To reduce the possibility of selection bias, the distribution of the participants was adjusted to be similar to nationwide statistics in Japan. Another limitation is the potential of recall bias, because parents who answered that their youngest child had received a seasonal influenza vaccine could remember a recommendation by a pediatrician or school/nursery more than parents who did not have their youngest child vaccinated. This research did not examine the information from the schools/nurseries in detail, nor assess information from web sites or social networks (family, friends). These information sources may influence parents’ knowledge and decision-making [24]. This study also did not assess parents’ attitude and beliefs [25]. Finally, we did not consider any vaccination-related costs, including respondents’ travel costs. Further studies should be conducted to consider this aspect.

## Conclusions

The main finding of the study is a significant association between household income and influenza vaccination of the youngest child in the household. Financial support for low income households might improve influenza vaccination coverage in children, because the cost could be a barrier to vaccinating children in low income households. Additionally, information from pediatricians and schools/nurseries could motivate parents to have their children vaccinated.
